# Propagandism of Dental Education in Our Schools

**Published:** 1907-10-15

**Authors:** George Zederbaum

**Affiliations:** Charlotte, Mich.


### PROPOGANDISM OF DENTAL EDUCATION IN OUR SCHOOLS.

GEO. ZEDERBAUM, D.D.S., CHARLOTTE, MICH.

Members of the Michigan Dental Association:—I do not know why I was asked to read a paper on this important subject, whether it was because I am somewhat interested in promulgating such teachings, or, what is more probable, because your program committee has a grudge against the Association. However, I am not to blame but have prepared what I could along the requested line.

It was my intention to give you a more exhaustive statistical report gathered and complied by myself; but, owing to the change of date of meeting to over a month earlier than usual, I am unable to do so. Before going into the details of my own experience, I intend to show what the dental profession is doing about this subject, and will quote various statistics and statements of men very prominent in our profession. There should certainly be no question in any of our minds as to the necessity for introducing dental education in our public schools, certainly not if one stops to study the various figures presented here.

In an address to the students of the Royal Dental Hospital of London, Dr. Osler, said; "You have just one gospel to preach, and that is the gospel of cleanliness of the mouth, the cleanliness of the teeth and the cleanliness of the throat. These three things must be your text throughout life. Oral hygiene—the hygiene of the mouth—there is not any one single thing more important to the public in the whole region of hygiene than that, and it is with that that you as practitioners will have to deal." Dr. Brown, of Chicago, says: "If the mouths of children in our public schools could be systematically examined by competent persons and instruction given and enforced with regard to the intelligent use of brushes and antiseptic solutions, the death rate of this country could be materially lessened and the percentage of illness much reduced, and a stronger and more vigorous race result in consequence of these prophylatic measures." Where do these men get such ideas?

It was found on examination that nearly 80 percent. of the children in Great Britain's Industrial Schools were suffering from decayed teeth. Dr. George W. Cook reports, after investigation of 220 mouths, that 171 contained the bacillus tuberculosis, thereby showing conclusively that even that dreaded plague may enter the system by way of carious dental organs. And, by the way, are not the avenues of infection of most specific diseases by way of the alimentary canal, and especially so where unhygienic, putrefactive conditions of the mouth exist? In the third annual report of the city of Strasburg, of 2269 children examined between the ages of three and four, only 362 had good teeth—less than 16 percent. Of 2,103 between the ages of six and eight, only 160 or 7½ percent, had good teeth, and it goes on to state that this third annual report shows a remarkable improvement in general health of the public school children, less headache, earache and stomach trouble. I wonder what must have been the condition there prior to the introduction of compulsory education in the care of the teeth? A further report from same source states that in one school of 700 pupils, 500 between ten and eighteen years of age, fifty cleaned their teeth, 275 used a brush sometimes, 175 did not even own one, and of 200 in the primary departments, between the ages of six and ten, only ten had a brush. It was this alarming condition of decay that caused the erection of a Dental Hospital there, for the exclusive use of school children, and every child is subjected to a dental examination on entering school, and twice per year thereafter until the age of thirteen. In Germany of 20,000 children between the ages of six and sixteen, 95 percent. had dental caries in alarming proportion. In Freiburg there is established a municipal school dental clinic, the faculty of which examines the teeth of all the children, whether school children or not. Each child is furnished with a card bearing the history of the condition of his teeth on the date of examination, and on the back of the card are printed instructions for the care of the teeth. The parents of children found to have defective teeth are notified and at the same time are requested to send their children to the dental clinic for treatment at the expense of the city. In England 75 percent. of all the children are sufferers from unhygienic conditions of mouths. In Frankfort, of 1,020 children, (482 boys and 538 girls) examined, there were found 28,673 defective teeth. Boys had 12,326 and only 2,116 sound, and 15,746 of girls teeth were defective with only 931 sound ones. Two hundred and ninety-three girls or half of the total number were suffering in the general health in consequence of decayed teeth. These figures go to show that 90 percent. of all the teeth examined were defective. In Paris both medical and sanitary inspection is obligatory in all schools, and, by virtue of the municipality the pupils have their teeth examined every six months. In Stokholm, in obedience to King's command, there is systematic examination of mouths of all school children since 1899. While I am far from being an advocate of monarchial powers, I think this a good thing in this particular instance and it is almost deplorable that our president has not the same authority. The examination of 3,156 school children in New South Wales, in all of 73,708 teeth, showed 16 percent. of the permanent and 24.47 percent. of the temporary teeth in carious condition. In all, 94 percent. of these children had decayed teeth. In the British Army, during one year, 4,740 men had 7,604 teeth drawn—just think of the sacrifice! Out of 23,000 men refused enlistment from all causes in one year, 5,000 men were rejected for either the loss or on account of decayed teeth. In reply to my question, I have the report of the Surgeon-General of the United States Army, that in the fiscal year 1905 to 1906, 18,000 young men were rejected for all causes and 1,000 were refused enlistment on account of bad teeth alone.

An English writer recently pointed out very strikingly the importance of oral hygiene and his words may be quoted here to an advantage: "I am convinced that a septic condition of the mouth tolerated in ordinary health, becomes a source of great danger of the patient during an acute attack of specific illness, such as typhoid fever, ulcerative endocarditis or pneumonia and may be an important issue of the case." Another distinguished specialist asserts (says Prof. W. D. Miller), that loss of appetite, nausea and general ill health, may be brought about by want of proper attention to the mouth, causing a chronic state of putrefaction, the products of which are absorbed by the mucous membrane our progressive minds are unanimous in the belief that no with serious results to the general health. The examination of 987 children developed that 99 percent. of all those suffering from the caries of the teeth were affected with putrefactive condition and swelling of lower glands, of which no physician would be able to make a diagnosis."

Again, Dr. Brown, in his address before the American Medical Association, said in part: "The presence of bacteria in such a great variety and number in the mouth at all times, must be looked upon as a menace not only to the teeth in their relation to the dental caries, but through their action as well upon the mucous membrane, rendering it more susceptible to the germs of specific fevers, and upon the digestive tract, for many complaints of the stomach and intestines have been found to be caused by mouth bacteria and their products. Even the lungs are subject to this influence from the mouth. Therefore its thorough disinfection becomes a matter of first importance." Dr. Burton Lee Thorpe, says that the mouth is the breeding place of the bacillus influenza and that oral sepsis is the predisposing cause of influenza and that those with hygienic mouths are practically immune from it.

From the above conclusions it will be readily seen that our progressive minds are unanimous in the belief that no person's health can be better than his teeth. It has been well demonstrated that the education of a country must begin first, in its schools; and, true to this, we find men are awakening to the fact and are establishing educational institutions, and are lecturing, with a great sacrifice to themselves in public schools, and, so to speak, are opening the long shut eyes of the laity to the urgent need of a clean, healthy vestibule to the human system. And so it is that we read that since the experiment in the city of Strasburg, there are more than a dozen cities in Germany that have established like dental clinics for compulsory examination and care of the school children's teeth. The British army council, in view of the enormous loss of teeth in its ranks commenced supplying the soldiers with artificial dentures, and were obliged to discontinue this experiment owing only to the fact that the soldiers so treated would not live up to their agreement to pay for such services out of their monthly allowance of 25 cents, and when the military authorities made demands for payment, Tommy Atkins decamped, teeth and all. Russia, that supposedly uncivilized and backward country, is beating us in the very line in which we know we ought to be the stronger. Even there they have established dental clinics in various cities for the care of school children's mouths. In St. Petersburg, alone, a city of just a trifle over a million inhabitants, a city the size of Philadelphia, there are nine such dispensaries. The New York Association for the improvement of condition of the poor realizing after examination of school children under their jurisdiction that there were 18,000 of them needing dental attention have established dental infirmaries at an annual expense of $3,000 where these children are taken care of, and not by students, but, by first-class dental practitioners. In Cleveland there is established a dental clinic in the city infirmary for the exclusive care of 1,500 children of the poor. In Malden, Mass., in spite of the failure of passage of the bill for compulsory examination and care of the teeth of the school children, there is started a strong movement in this direction and the examination and care of the children's teeth is an assured fact.

We read that a movement is on foot in Chicago to establish a $300,000 school, the express mission of which will be the salvation of the human stomach. "A most excellent move," said one of our professional savants, "for no man can feel better than his stomach." But the greatest factor in abusing this vastly important organ is the utter neglect of keeping the teeth in proper condition, and the success of this institution will depend largely upon the stress placed on oral hygiene and the preservation of the individual's teeth. We read further, that, in the Chicago Hospital a special clinical department is to be added, in which a regularly established system of mother's clinics for the free instruction of parents in the care of children will be held. It is hoped that mothers will be given, along with the rest, a thorough instruction relative to the children's teeth, and then no mother can say she did not know this or that about her child's dentition. The enthusiastic and scholarly Dr. Raylor of Cambridge has offered to defray the cost up to $250 for a years attention to the teeth of children attending the schools of his town. A very commendable offer, and I wish there were more such benefactors.

Now, then, we come to the question—do members of the medical profession who are constituents of the various Boards of Health, do their duty by themselves and to the public if they do not insist upon systematic care of children's teeth? Most emphatically, no! Take our own State Board of Health for instance—this board publishes, "for the general dissemination of sanitary knowledge," quarterly pamphlets dealing with prevention of certain specific, epidemic diseases. One pamphlet is on diptheria and its restriction and prevention; one on tuberculosis; one on typhoid fever and its restriction and prevention, etc.; and one just of recent issue, deals totally with the sanitary teaching in our public schools. A most commendable work indeed, but let us stop a minute —out of sixty printed pages we find but a few lines that at all mention the care of the teeth, while we find a whole paragraph on the care of eyes and ears. And is it not a proven fact that most earaches are in reality toothaches? I give full credit to Prof. Ferris, who, though not a medical man, recognizes the necessity of cleansing the teeth and in his article in the aforementioned pamphlet urges regular visits to the dentists. I say it is within the jurisdiction of the Board of Health to insist on dental care and dental education in our schools and then and only then can we expect the fathers and the mothers to come to know whether their child has his second tooth. In most of our schools physiology and hygiene are taught, to some extent, and we will agree that the dental hygiene should find its way into the school curriculum as well. Just imagine, gentlemen, of the immense progress we would make if the 18,000,000 children in our schools to-day should receive the benefit of such instruction.

Only recently, in Ann Arbor, a Miss Ellis of Grand Rapids was highly praised for the eloquent appeal before the Michigan Schoolmaster's club for a better parental and scholastic training of the young in the rules of health and hygiene. This lady, declared that personal hygiene was sadly neglected and attributed much of disease and the accompanying evils to the ignorance of its laws. She insisted on practical teaching of all matters pertaining the elevating the bodily health, and went even so far as to recommend elementary lectures in bacteriology with particular study of tuberculosis and its prevention and cure. I am sorry that one thing was left out of her lecture—the unclean, diseased condition of the mouth. Only a few weeks since, at the Tuberculosis Convention at Washington, the celebrated Dr. Dunn of Boston stated that the terrible white plague increases from month to month and year to year, and that, in early life tuberculosis does not affect the lungs as it does in the case of adults, but frequently remains hidden in the internal glands in children, until an acute form of tubercular meningitis or pneumonia causes death. "Children," he declared, "have no power of resistance against acute outbreaks," and he said, also, that this disease entered the body through the lungs, through the intestines or through the tonsils and larynx and located itself in the glands. As the mouth is the very entrance to all the passages, is it not important to teach oral hygiene, thereby guarding the gates of admission and lessen the tendency or susceptibility and reduce the predisposing cause to infection.

The problem of educating the school children to the necessity of oral hygiene and the care of the teeth is a hard one, to be sure. I do not stand alone in my opinion that our medium is through the school and, while attempts have been made by progressive dental practitioners in Missouri, Mississippi and Massachusetts, to pass laws making it compulsory to teach these subjects in the schools, these attempts, perhaps from the lack of enthusiasm, insufficient support or indifferent perseverance, or perhaps from the lack of the whole three, have proved unsuccessful.

Before proceeding with this educational propoganda I outlined my course and have altered it only to suit the convenience and conditions. Here is what I have done: I examined in all 500 mouths of school children between the ages of six and sixteen and found out of that number 450 in need of dental attention. All of the various dental branches, with the exception, perhaps, of plate making, were found to be called for. Here were numerous cases for treatments, for fillings and many for the extracting specialists and oh, my! what a field for the orthodontionist! In aggregate there were 2,700 teeth that needed attention, thus bringing the average of six teeth to each mouth. Let us see for a minute what time it would take to place the mouths of school children in good condition in just one county. It is estimated that Eaton County has to-day some 3,000 pupils, at the given average there would be in the neighborhood of 2,700 children in schools needing dental work. Now, at six teeth per capita, there would be 16,200 teeth to take care of. I wonder if any of you have an idea what this enormous number means? Polk's Dental Register gives the number of dental practitioners in my county as twelve.

Let us suppose that ten of these practitioners are willing to give their time to the care of these teeth, working and completing five teeth each per day; at this rate of fifty teeth completed each day, it would take these ten practitiones working daily, a whole year to place these children's teeth in good condition. And gentlemen, this is not all; in the county mentioned there are some 576 square miles and there are 136 widely distributed schools to visit, and the roads are not of the best, and the weather is lots of times severe, so considerable time is lost on these accounts. You see the undertaking is of a large magnitude and would require co-operation on the part of our profession and the teachers. It is with these ideas in mind that I wrote Dr. Hoff about a year ago, and received from him a letter of recommendation to our Board of Education, and it was but a little while when a committee called on me asking me to deliver a lecture before the State Teachers' Institute. Accordingly I appeared on the program and quote from a clipping merely to show that the sentiment is with us, and the people and the press are willing to assist us. It said: "Dr. Zederbaum gave a half-hour lecture before the Teachers' Institute. It was full of interest and was much appreciated by all present. His topic was "Oral Hygiene and Prophylaxis" and his arguments in favor of a thorough teaching of youngsters as to the care of teeth, was sound. He predicts compulsory training in the schools in this country within a few years. He gave histological as well as physiological structure and function of the teeth. Explained cause and prevention of decay and ended by asking the co-operation of all the teachers in promulgating the cleanliness about the mouth. He expects to deliver lectures in various schools during the coming season and we are sure his work along this line should be encouraged." By this first lecture, you will readily see, I have gained a sort of perfunctory acquaintance with many of the teachers and while they in themselves are far from being efficient in oral hygiene, their enlistment in our ranks will prove for the best. I heartily agree with

Dr. Byington (*Dental Review*, Nov. '06), that a plan should be adopted whereby the Governor of each state should select well qualified dentists to lecture at least once each school term, impressing the pupils with the importance of dental hygiene. I would further suggest that these lecturers appear before the various school teacher's conventions, institutes and clubs, and enlighten them as well and, further, to ask their co-operation, and to insist upon their pupils by example as well as precept, if only for the sake of beauty, to keep their teeth and mouths clean.

My lectures before the children were in as plain language as I could put it and still give comprehension. I used Dr. Hyatt's little booklet entitled: "The teeth and their care," as a guide, and with its help outlined the subjects which I wished to present to them. They were: physical and chemical structure of the teeth. Eruption of the temporary and then the permanent teeth. Explained the prevalent cause of irregularities of the teeth, showed the cause of decay and its influence upon the general health, on breath and beauty. Mouth breathing and thumb sucking; explained in a simple manner on the black board what makes the tooth ache, and what is generally meant by the so-called "gum-boil" and explained the manner of some of the simpler operations for cleaning the teeh for the purpose of educating the majority, that had never been in a dental office, and had never even owned a tooth brush. I also showed the manner in which the teeth should be brushed, and how the irrigation of the mouth and gargling of throat should be accomplished. The lecturing over, my young friends were not so timid when I called them by name and examined each mouth, and noted all diseases and irregularities, the result of the examination of each tooth was marked out on a chart. Here is a copy of the blank form I prepared:

Name of Pupil--------------------Age (nearest birthday)-------------
Name of Teacher----------------------District----------------------
Name of Father (if not living, give mother's)--------------------------
General condition of health of pupil--------good--------fair------bad.
Has the pupil ever had any dental work done (extracting excepted)?

Yes_______________________________No___________________________
Does the pupil own a tooth brush___________________________Yes or No.
Does the pupil use it_________________________________________Yes or No.

---

Date of Examination_____________________________________________
Were parents or guardians present__________________________________

CHART OF THE TEETH.

| | | | | | | | | | | | | | |
|---|---|---|---|---|---|---|---|---|---|---|---|---|---|
| 7 | 6 | 5 | 4 | 3 | 2 | 1 | 1 | 2 | 3 | 4 | 5 | 6 | 7 |
| G | F | E | D | C | B | A | A | B | C | D | E | F | G |

Remarks_________________________________________________________
Scaling and cleaning__________________Regulating_____________________

---

I might add here, that, when I was ready for my work, I wrote the teacher of a certain school and explained my motive, and asked her or him, as the case might be, to interview the Board of Trustees and see whether such a lecture and examination of the mouths as I proposed, would be agreeable. I am glad to state that the trustees of only one school out of eleven thus addressed, refused permission, thinking perhaps this was another one of the many schemes for personal gain. When permission had been granted, I sent the Teacher as many blanks as there were pupils under her charge, and asked her to fill out all above the double line and have the whole ready for me on the appointed day. Of course it takes a lot of time to examine even twenty-five mouths, so to economize time, my wife accompanied me on these tours of inspection and marked the charts as I went along in each individual mouth. True, some of the bashful ones among these youngsters, would occasionally object to being examined; but some of them changed their minds, however, when I gave out samples of tooth powder and mouth washes furnished me by the manufacturers. Others still persisted, only to tell me secretly that their mouths were in such a horrible state that they would not have anyone look into them. Really, gentlemen, if there be less talk about keeping the school room more ventilated and more attention be paid to the cleanliness of the mouths, the air in these rooms would not become vitiated so speedily as to require constant opening and shutting of windows. True, God's air is pure and cheap and we want lots of it, but,

I doubt whether it gets into the lungs as pure as it was intended it should, when it has to pass such foul mouths as I had the occasion to examine. The scheme of having the teacher fill out the blanks, worked admirably and saved a lot of time. All the questions asked on blank forms were, in my mind, necessary for the future reference. I hope sometime to cover same ground again and see for myself what difference was made in condition of these mouths.

Now, Gentlemen, I trust I have proven to you the necessity for a propagandism of dental education in our public schools, and the plan similar to that on which I have worked, would, I think, prove satisfactory. Now then comes the question how shall we do it, how shall we carry this propoganda into effect; This morning there came to me a pamphlet from a certain commercial dental toilet manufacturing concern, of an educational association, and, gentlemen, there are many points in that advertisement that appealed to to me as rational, and such as together with a few other suggestions will certainly accomplish the desired end. So, now that our eyes are opened to the situation, let us not ponder over it, but take this matter up as a body, and act in harmony and in unison to accomplish the greatest good for humanity that our profession is capable of doing.

DISCUSSION.

Dr. R. W. Bunting: Dr. Zederbaum informed us that he had not had time to look up the necessary statistics. I wonder what he would have given us if he had been given more time. It is a subject I am very much interested in myself because I have had a little dabble in it.

I wish very much he had let me know what line his paper was to have followed because I think I could have brought some statistics to show the result of my work. I have had the opportunity of examining 1,500 children's mouths with Dr. Bean in the public schools of Ann Arbor, and out of that number of mouths we have compiled records and charts which have been made up more especially for statistical advantage. They are not completed as yet, and I am working diligently on them to get some reliable data. We have so far not been able to get anything more than the bare results completed. There are two or three things coming out very strongly to me, unless I am mistaken. In our examination we divide the children into three classes, good, fair and poor. We cannot be sure how many times a child cleans his teeth. I asked one boy if he had cleaned his teeth, he said no, not that week. I told him I thought he had better do it sometime during the week. Sometimes they say they do when they don't do it. I find of all the school children I examined at Ann Arbor that the percentage of decayed teeth is very low, only 6 percent. I was surprised to see how well most of the children kept their teeth, and how well they seemed to understand the care of them. In the mouth of one child, a tooth was out of shape, and I said to her something should be done for that and she said her mother had taken her to the dentist about that and he is going to take care of it. I believe in this education of the young people to properly take care of their teeth. In the public schools in Ann Arbor the children are taught something about hygiene generally and about the teeth. When I saw how ready the teachers were to take this instruction up, how ready they were to have those children told about the conditions it seemed a pity that somebody wasn't ready to go and tell them. I think the teachers would heartily co-operate with us.

DR. SHATTUCK: I am surprised at these figures shown by Dr. Zederbaum. In Detroit out of 300 that I examined in April of this year there were ninety-eight needed dental operations. I think the percentage is much lower than the essayist has placed on the blackboard.

DR. LOEFFLER: All the year I have been very much interested in the work of Dr. Bunting. I am also greatly pleased that we have such men as Dr. Zederbaum who give up their time for finding out these things for us. I wish more of us would do this kind of work. I think too many of us are after the almighty dollar, and leave it to a few to solve such problems. Naturally we dentists want to know what there is in it in these things. We must start with the children and educate them along these lines; they might be compelled at least once a day to clean their teeth. The teeth once put in right condition, a proper habit formed, they are apt to be kept. So this is a work that I think our lady dentists ought to take up; but it should appeal to the men as well.

